# Supplementary material for: Quantifying collective interactions in biomolecular phase separation
Source: Nat Commun. 2025 Aug 19;16:7724. doi: 10.1038/s41467-025-62437-y (PMC12365266; doi:10.1038/s41467-025-62437-y)
Supplement: Supplementary file 1 — Supplementary Information [file 41467_2025_62437_MOESM1_ESM.pdf]

Supplementary Information

Quantifying collective interactions in biomolecular phase separation

Hannes Ausserwöger[1,*], Ella de Csilléry[1,*], Daoyuan Qian[1,*], Georg Krainer[1,*], Timothy J. Welsh[1], Tomas Sneideris[1], Titus M. Franzmann[2], Seema Qamar[3], Nadia A. Erkamp[1], Jonathon Nixon-Abell[3], Mrityunjoy Kar[4], Peter St George-Hyslop[5,6], Anthony A. Hyman[4], Simon Alberti[2], Rohit V. Pappu[7], Tuomas P. J. Knowles[1,+]

[1] Centre for Misfolding Diseases, Yusuf Hamied Department of Chemistry, University of Cambridge, Lensfield Road, Cambridge CB2 1EW, United Kingdom

[2] Biotechnology Center (BIOTEC), Center for Molecular and Cellular Bioengineering (CMCB), Technische Universität Dresden, Tatzberg 47/49, Dresden, Germany

[3] Cambridge Institute for Medical Research, Department of Clinical Neurosciences, Clinical School, University of Cambridge, Cambridge, CB2 0XY, United Kingdom

[4] Max Planck Institute of Cell Biology and Genetics (MPI-CBG), 01307 Dresden, Germany

[5] Department of Medicine (Division of Neurology), Temerty Faculty of Medicine, University Health Network, University of Toronto, Toronto, Ontario M5T 0S8, Canada

[6] Department of Neurology, Columbia University, 710 West 168th Street, New York, New York 10032, USA

[7] Department of Biomedical Engineering and Center for Biomolecular Condensates, Washington University in St. Louis, St. Louis, MO, USA

[8] Cavendish Laboratory, Department of Physics, University of Cambridge, JJ Thomson Road, Cambridge CB3 0HE, United Kingdom

* Contributed equally

[+] Correspondence should be addressed to tpjk2@cam.ac.uk

Supplementary Figures

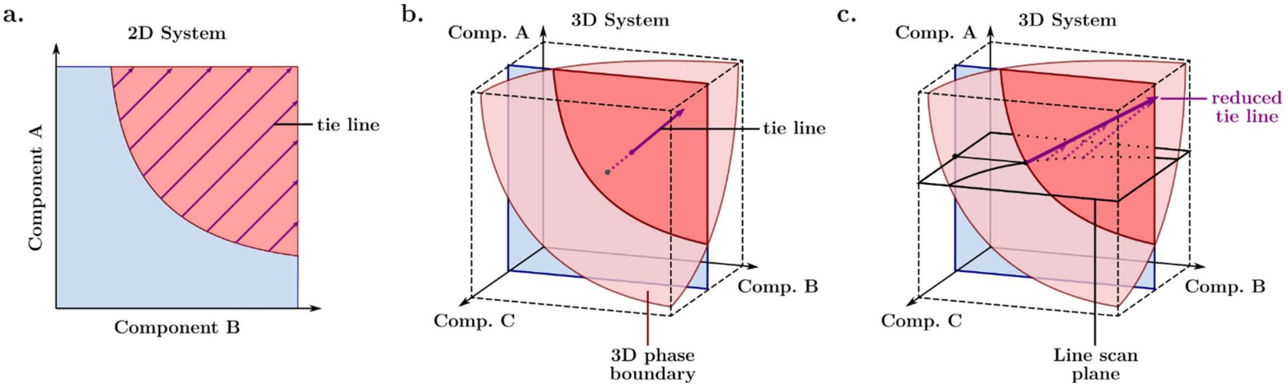

Supplementary Figure 1. Schematic illustration of tie line reduction to the 2D measurement space. (a) In a pure 2 component system, tie lines are constrained to the 2D measurement plane only. Thereby the reduced tie line gradient is equivalent to the 'higher' dimensional tie line. **(b)** Upon partitioning of additional components, not included in the measurement plane, tie lines become higher dimensional objects[8]. **(c)** Approximate information of the higher dimensional tie line gradient with respect to the measurement plane of interest can then be extracted from tie line reduction. The reduced tie line then represents the intersections of tie lines originating from a fixed component A dilute phase concentration with the measurement plane[8].

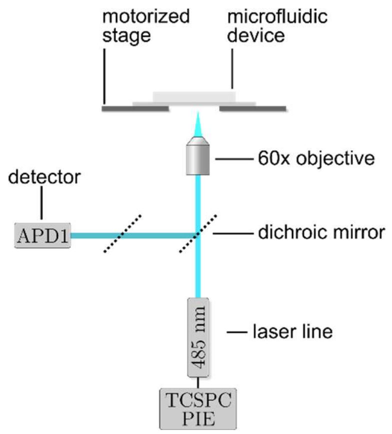

Supplementary Figure 2. Schematic representation of the custom-built confocal measurement setup. The setup consists of a 485-nm excitation picosecond-pulsed laser line, operated in pulsed interleaved excitation (PIE) mode. The laser light excites molecules in a microfluidic device through a high magnification objective. Emitted photons are spectrally separated and then detected by avalanche photodiodes (APDs) and time-correlated single-photon counting (TCSPC) electronics.

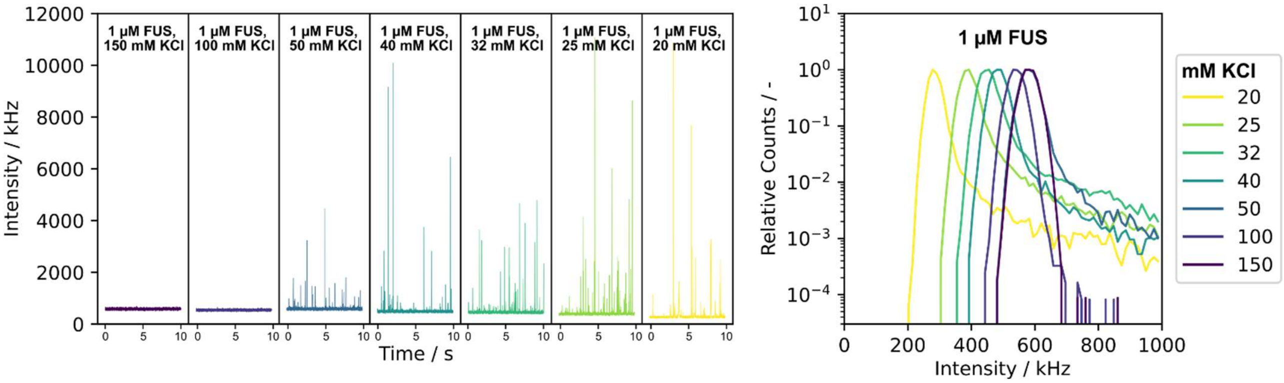

Supplementary Figure 3. Time trace and intensity histogram line scan data for FUS/KCl. (left panels) Representative individual intensity time traces for FUS at 1 uM (2 uM shown in main text Fig 2.) and varying KCl concentrations recorded using a microfluidic flow cell connected to confocal detection unit. Time traces show appearances of larger numbers of intensity bursts and decrease in the baseline intensity due to phase separation. Time traces measurements were performed in triplicates with additional repeats not shown (see SI Fig. 12 for exemplary variation between devices). **(right panel)** Intensity histograms of recorded time traces, with the maximum of the distribution representing the dilute phase concentration. These individual maxima are plotted against the total KCl concentration to give dilute phase line scan data.

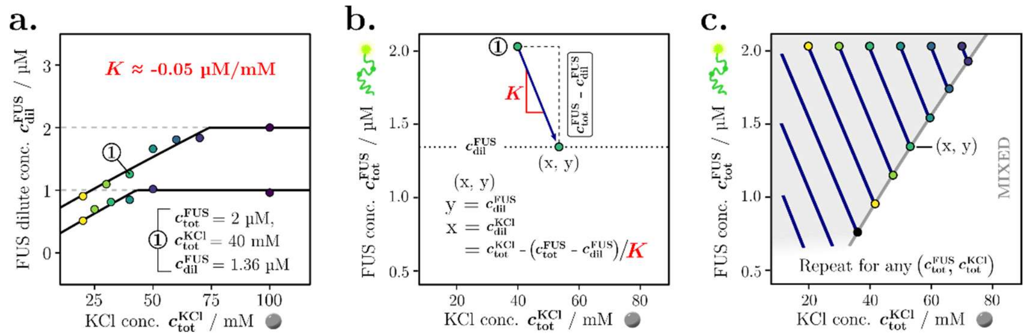

Supplementary Figure 4. Phase boundary extrapolation from dilute phase concentration response functions on the example of FUS/KCl. (a) To extrapolate the phase boundary the experimentally determined dilute phase concentration response functions and tie line gradient are used. **(b)** A point on the phase boundary is then identified by intersecting tie lines and dilute phase concentration of a given total concentration point (1). **(c)** This process is repeated for a set of predicted points using the experimentally determined dilute phase response function.

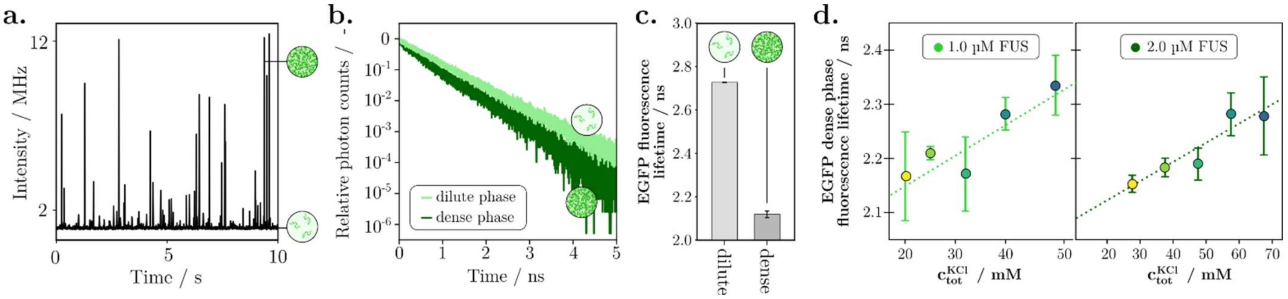

Supplementary Figure 5. Dense phase fluorescent lifetime determination. **(a)** Intensity time trace of 20 mM KCl and 1 µM FUS sample injected into microfluidic channel connected to confocal detection unit. Intensity bursts stem from the protein condensed phase, whereas the dilute phase forms a stable baseline. **(b)** Histogram of photon arrival times post excitation for dense and dilute phase signals, with the dense phase showing a faster decay. **(c)** Comparison of calculated dense and dilute phase fluorescent lifetimes from **(b)**, mean and SD are determined by fitting to the said decay slope. **(d)** Dense phase fluorescence lifetimes with changing KCl concentrations at both 1 and 2 µM FUS. Data are presented as mean ± SD from n>3 repeats.

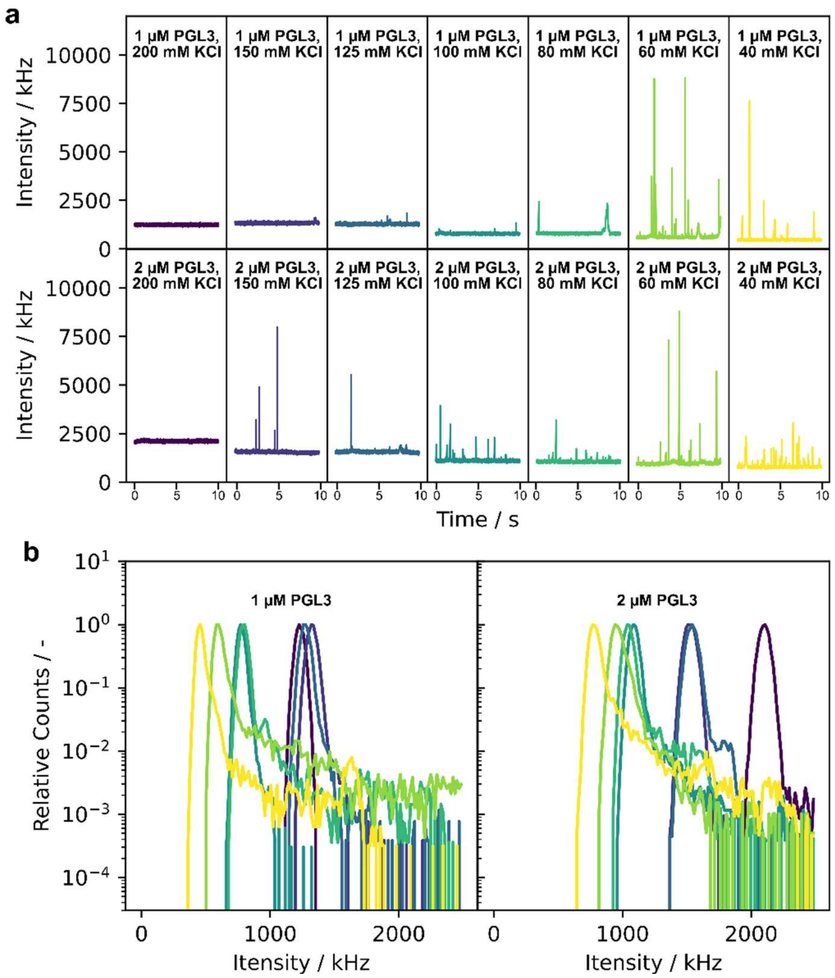

Supplementary Figure 6. Time trace and intensity histogram line scan data for PGL3/KCl. (a) Representative individual intensity time traces for PGL3 at 1 and 2 uM and varying KCl concentrations. Time traces measurements were performed in triplicates with additional repeats not shown. **(b)** Intensity histograms of recorded time traces, which are used to construct dilute phase line scan data at 1 and 2 uM PGL3.

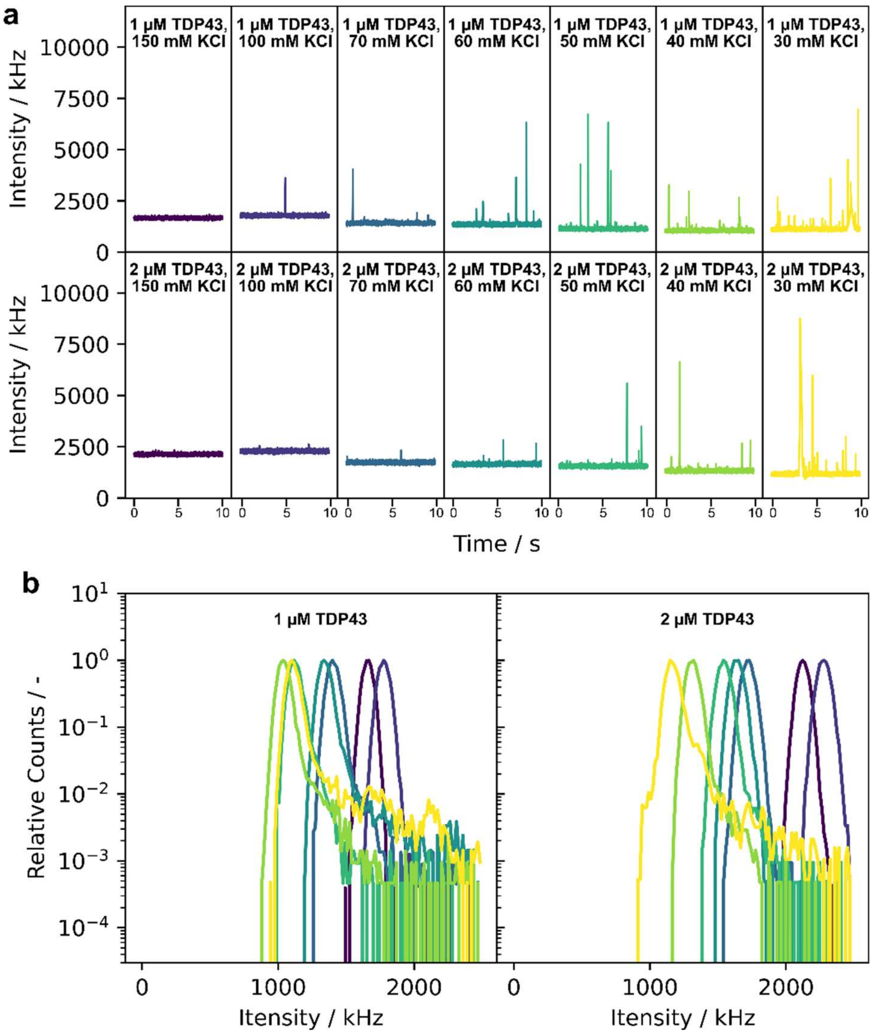

Supplementary Figure 7. Time trace and intensity histogram line scan data for TDP43/KCl. (a) Representative individual intensity time traces for TFP43 at 1 and 2 uM and varying KCl concentrations. Time traces measurements were performed in triplicates with additional repeats not shown. **(b)** Intensity histograms of recorded time traces, which are used to construct dilute phase line scan data at 3 and 5 uM TDP43. Laser settings were applied independently to the 1 and 2 uM concentration series in this case and accordingly the concentration calibration were performed separately.

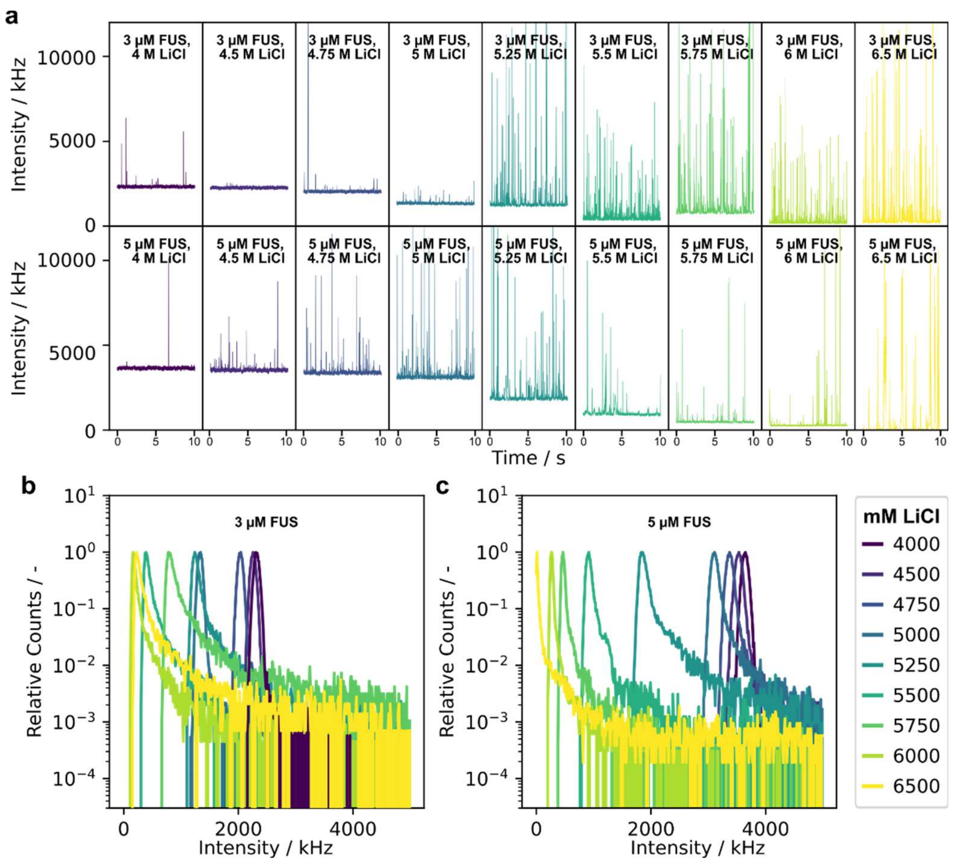

Supplementary Figure 8. Time trace and intensity histogram line scan data for FUS/LiCl in the high salt reentrant regime. **(a)** Representative individual intensity time traces for FUS at 3 and 5 uM and varying LiCl concentrations. Time traces measurements were performed in triplicates with additional repeats not shown. **(b, c)** Intensity histograms of recorded time traces, which are used to construct dilute phase line scan data at 3 and 5 uM FUS.

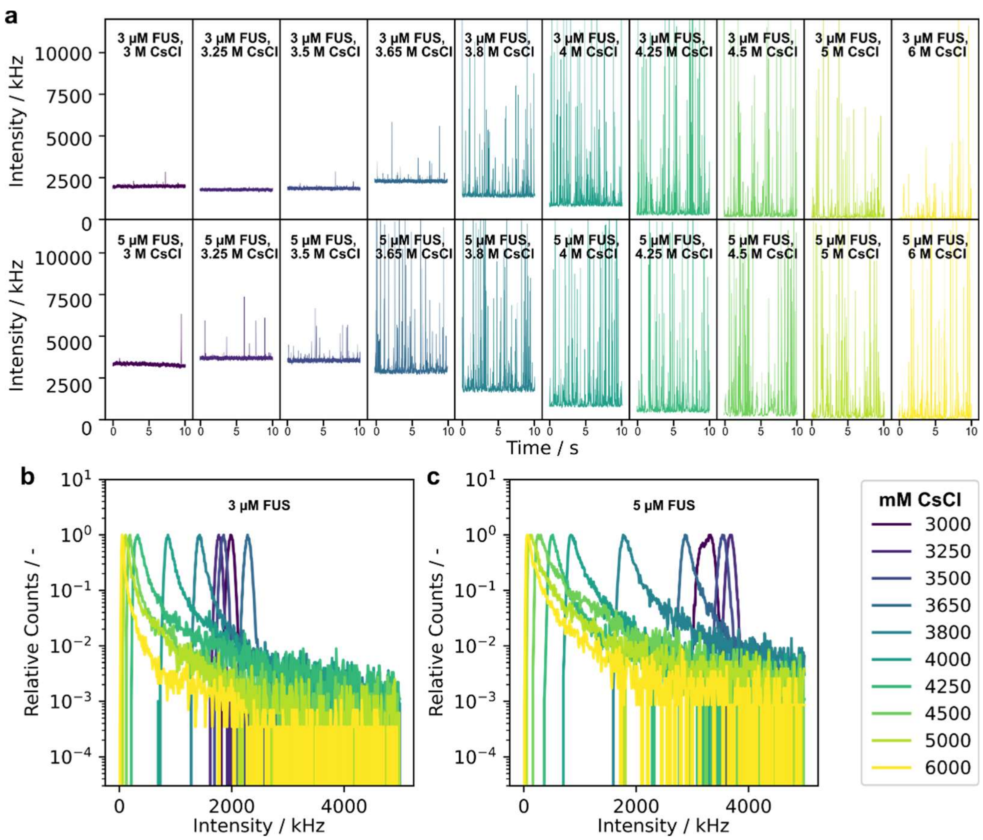

Supplementary Figure 9. Time trace and intensity histogram line scan data for FUS/CsCl in the high salt reentrant regime. (a) Representative individual intensity time traces for FUS at 3 and 5 uM and varying CsCl concentrations. Time traces measurements were performed in triplicates with additional repeats not shown. **(b, c)** Intensity histograms of recorded time traces, which are used to construct dilute phase line scan data.

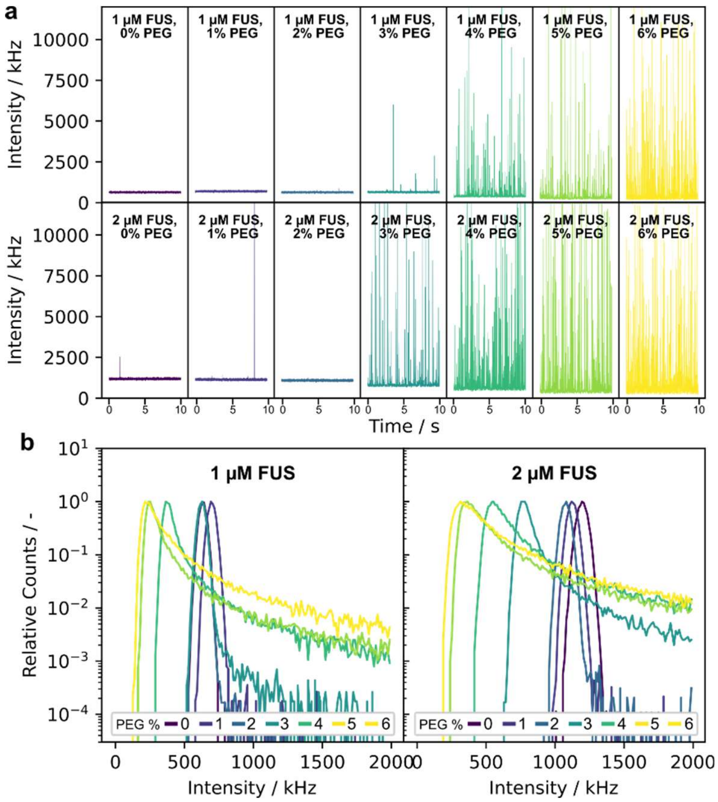

Supplementary Figure 10. Time trace and intensity histogram line scan data for FUS/PEG. (a) Representative individual intensity time traces for FUS at 1 and 2 uM and varying PEG concentrations. Time traces measurements were performed in triplicates with additional repeats not shown. **(b)** Intensity histograms of recorded time traces, which are used to construct dilute phase line scan data.

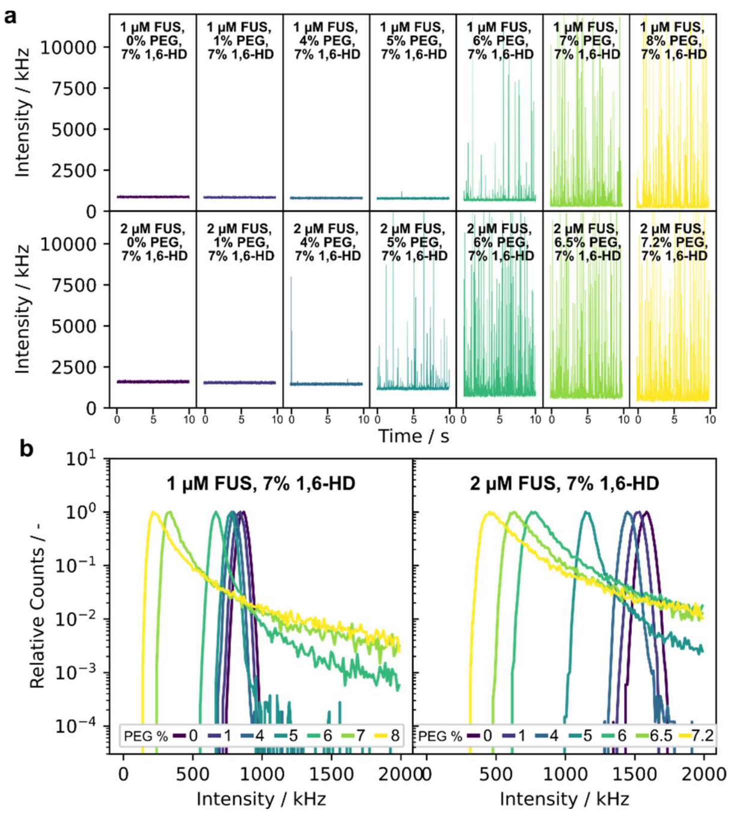

Supplementary Figure 11. Time trace and intensity histogram line scan data for FUS/PEG in presence of 1,6-HD. (a) Representative individual intensity time traces for FUS at 1 and 2 uM and varying PEG concentrations at 7 (w/v)% 1,6-hexanediol. Time traces measurements were performed in triplicates with additional repeats not shown. **(b)** Intensity histograms of recorded time traces, which are used to construct dilute phase line scan data.

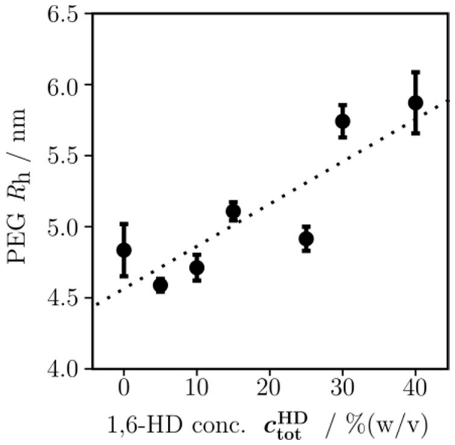

Supplementary Figure 12. PEG expansion under influence of hexanediol. Changes in hydrodynamic radius R_h of fluorescently labelled PEG (20 kDa) at 0.1 (w/v)% as a function of increasing 1,6-HD concentration. Data are given as mean and SD as determined from n = 3 independent experiment repeats.

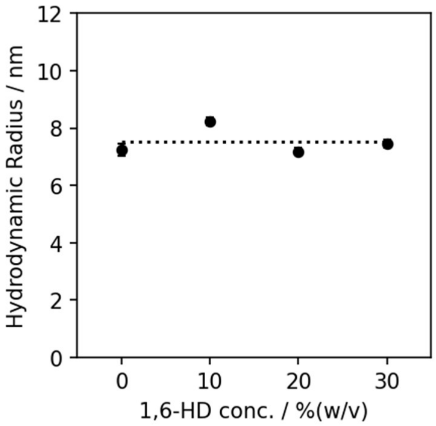

Supplementary Figure 13. G3BP1 hydrodynamic radius change under influence of hexanediol. Changes in hydrodynamic radius R_h of EGFP-tagged G3BP1 (1 u) as a function of increasing 1,6-HD concentration. Data are given as mean and SD as determined from n = 3 independent experiment repeats.

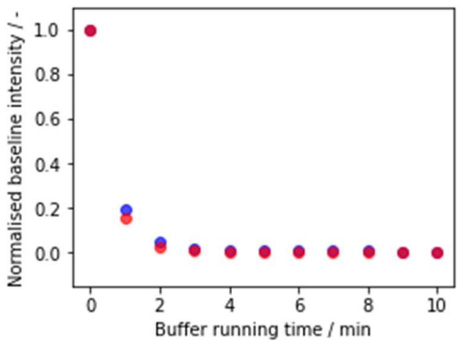

Supplementary Figure 14. Analysis of necessary channel equilibration times after the change of sample. Baseline intensity in the blue-emitting (blue) and red-emitting (red) channels. Shown is the normalized for the maximum intensity at the start of washing versus the runtime of the buffer washing. The microfluidic channel was first equilibrated with 1 µM FUS, 10 nM GUG aptamer and 8% (w/v) PEG at 50 mM TRIS (pH = 7.4), 150 mM KCl and subsequently flushed with 50 mM TRIS (pH = 7.4), 150 mM KCl at 100 µL/h to estimate the necessary channel washing/equilibration time. After 3 minutes of buffer wash, 99.3 % of the initial total intensity are removed.

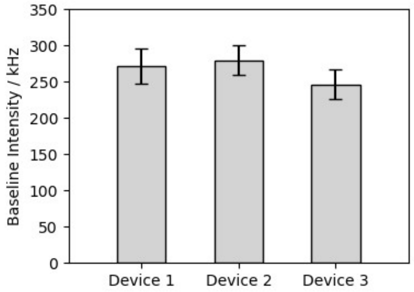

Supplementary Figure 15. Variations in dilute phase concentration measurements between devices. Baseline intensity of FUS at 1 µM and 5% (w/v) PEG in 3 different devices. Mean and SD are determined by fitting to time traces. Variations between devices are within measurement error.

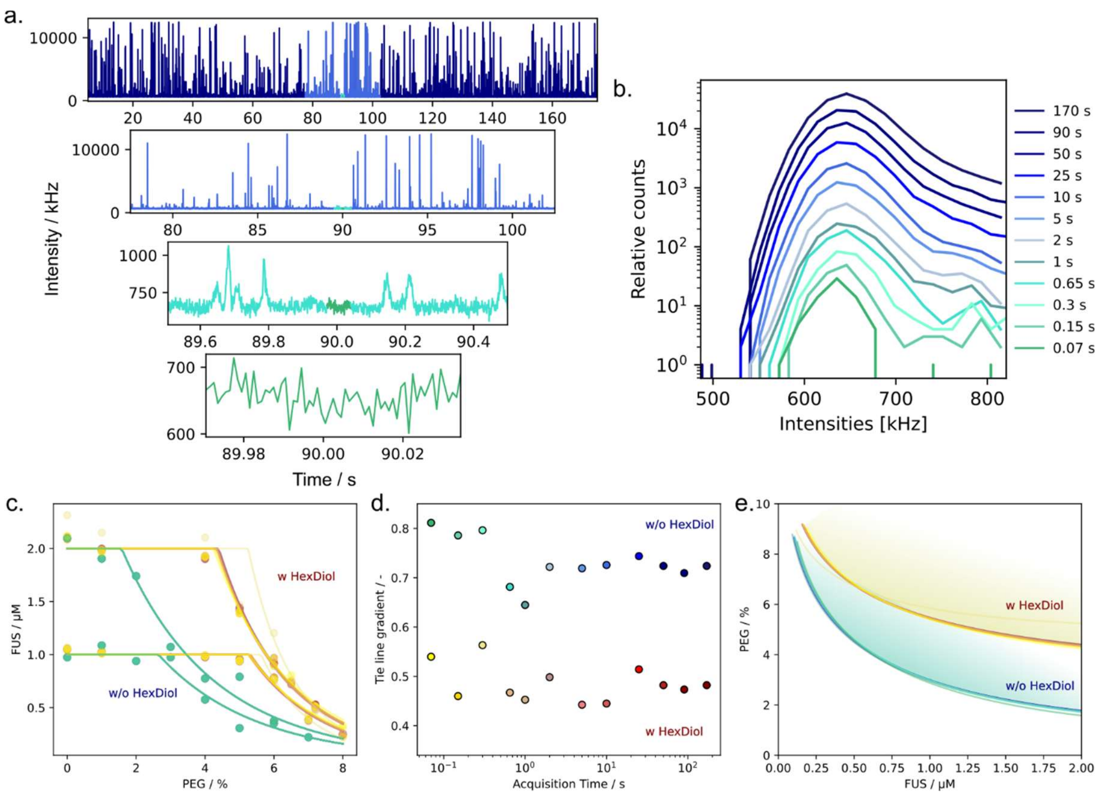

Supplementary Figure 16. Analysis of the necessary acquisition time for obtaining accurate readouts of dilute phase concentrations. **(a)** Zoom in of exemplary time traces acquired with confocal detection from total time spanning 180 down to 0.05 s. **(b)** Histograms of all intensity entries recorded in the measurement for decreasing total acquisitions times. **(c)** Overlay of FUS dilute phase concentrations obtained from different acquisition time windows for increasing PEG percentages. **(d)** Re-analysis of the tie line gradient of the FUS/PEG systems with and without 1,6-hexanediol using different total acquisition times. Color code: dark blue to turquoise and dark red to yellow depict the acquisition time axis **(e)** Overlay of phase boundaries obtained from different acquisition time windows.
